# Pattern of malaria transmission along the Rahad River basin, Eastern Sudan

**DOI:** 10.1186/1756-3305-4-109

**Published:** 2011-06-16

**Authors:** Yousif E Himeidan, Mervet M Elzaki, Eliningaya J Kweka, Muntaser Ibrahim, Ibrahim M Elhassan

**Affiliations:** 1 Entomology Unit, Faculty of Agriculture and Natural Resources, University of Kassala, New Halfa, Sudan; 2 Institute of Endemic Diseases (IEND), University of Khartoum, Khartoum, Sudan; 3 Tropical Pesticides Research Institute, Division of Livestock and Human Diseases Vector Control, P.O. Box 3024, Arusha, Tanzania; 4 Faculty of Medicine, Jazan University, Kingdom of Saudi Arabia

## Abstract

**Background:**

Understanding malaria vector mosquitoes and their infectivity dynamics is of importance in setting up intervention and control programmes. Patterns of malaria transmission have been shown to differ between non-irrigated and irrigated semi-arid areas of eastern Sudan. However, very little information is available regarding malaria transmission dynamics along the seasonal river's basin. Such information is required for the design of effective vector control strategies.

**Methods:**

A longitudinal study for mosquito sampling using pyrethrum spray catch (PSC) was conducted in two villages (Koka & Um Salala) along the Rahad River basin from December 2005 to October 2006. The *Plasmodium falciparum *circumsporozoite (CSP) and human blood index (HBI) were detected by ELISA. Three seasons were considered and the surveys represented cool dry, hot dry and rainy seasons were November - February, March - June, July - October, respectively. The CSP was compared between the seasons and populations using Chi-square test. The differences between the seasons and the populations in the other entomological indices, including Entomological Inoculation Rates (EIR), were measured using Tukey-Kramer HSD and Student T-test, respectively. The association between *An. arabiensis *density and monthly total rainfall was examined using regression analysis.

**Results:**

A total of 1,402 adult female anopheline mosquitoes were sampled, of which 98% were *An. gambiae *complex; the rest were *An. rufipes*. All specimens of *An. gambiae *complex identified by the PCR were *An. arabiensis*. Bimodal annual peaks of *An. arabiensis *densities were observed following the peak of rainfall and recess of the Rahad River after a time- lag of two months (Koka *r *= 0.79, d.f. = 1, *P *= 0.05; Um Salala, *r *= 0.88, d.f. = 1, *P *= 0.02). The CSP differed significantly among the seasons only in Koka (*P *= 0.0009) where the mean was nine times higher than in Um Salala (*P *= 0.0014). Active transmission was observed in Koka during the hot, dry season (CSP = 6.25%) and the EIR was observed to be 0.01 ib/p/n during this time. The EIR peaked to 0.71 ib/p/n during the rainy season and decreased to 0.18 ib/p/n during the minor peak of the cool dry season (*P *= 0.54). The combined annual average of the EIR for both populations was 55.48 ib/p/y and, typically, it would take approximately 192.7 days for an individual to receive an infective bite from *An. arabiensis.*

**Conclusion:**

The bimodal annual peaks and the active transmission observed during the hot dry season suggested low to moderate perennial malaria transmission pattern. Infectivity and transmission rates increased with proximity to the river following the peak of rainfall and the subsequent recession in the flow of the Rahad River. Current vector interventions can be integrated with larval control and should be formatted in accordance with targeted according to the time and space.

## Background

Variation in exposure to malaria-infected mosquitoes is almost certainly the main force behind focal malaria transmission [1-3]. An understanding of both the temporal and spatial variations in human biting rates and exposure dynamics has been used to create opportunities for focused malaria control [4]. However, targeting malaria intervention would require, in a particular region, a comparative understanding of transmission dynamics, which could be achieved through quantifying the potential risk factors. It is important when malaria transmission rate is on decline, that the control measures be directed towards the transmission foci [5-7].

The estimation of the entomological inoculation rate (EIR) provides a standard and relatively simple means of quantifying levels of human exposure to infected mosquitoes [8]. The EIR uses the proportion of mosquitoes containing sporozoites, and the human biting rate per unit time [9]. These indices that have been shown to be driven largely by environmental factors [10]. The EIR is therefore considered the more direct measure of transmission dynamics than the traditional measures of malaria parasite rate or hospital-based measures of infection or disease incidence [11-13]. Estimating the EIR also remains the most favoured measurement for assessing the effect of vector control actions because it quantifies the parasite-infected mosquito pool and its propensity to transmit infectious parasites to the human population [13]. There are, however, substantial gaps in the annual EIR across Africa, and past estimates of EIR were found to be available in only 23 of the 54 African countries, with 56% of the measures coming from only four countries (Kenya, Burkina Faso, Tanzania, and The Gambia) [14].

In eastern Sudan, the EIR estimates are only available in non-irrigated, rain-dependant agricultural areas. This region as reported where two to three infective bites per person per year occurring entirely at the end of the rainy season [15]. A different pattern of malaria transmission, from perennial to moderate transmission has been observed in irrigated semi-arid areas of eastern Sudan [16,17]. However, very little information is available about the seasonal variation and intensity of malaria transmission along the river basin. Such information is required for the design of effective vector control strategies. The present longitudinal entomological surveys examined seasonal variations of the EIR at two villages along the Rahad River basin in eastern Sudan.

## Methods

### Study area

**Figure 1:**
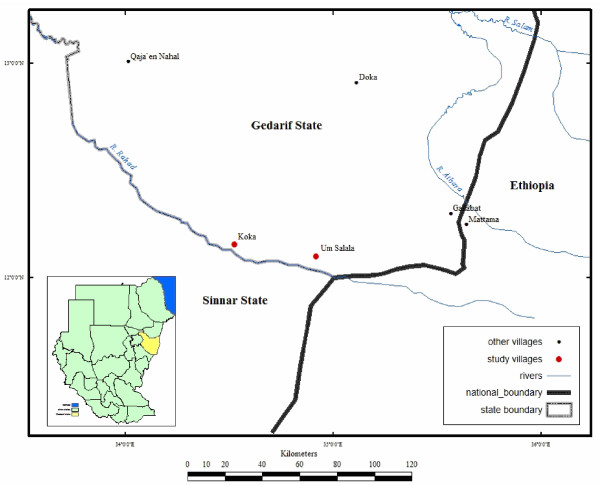
**Map showing the study villages in southern Gadaref state, eastern Sudan**.

**Figure 2:**
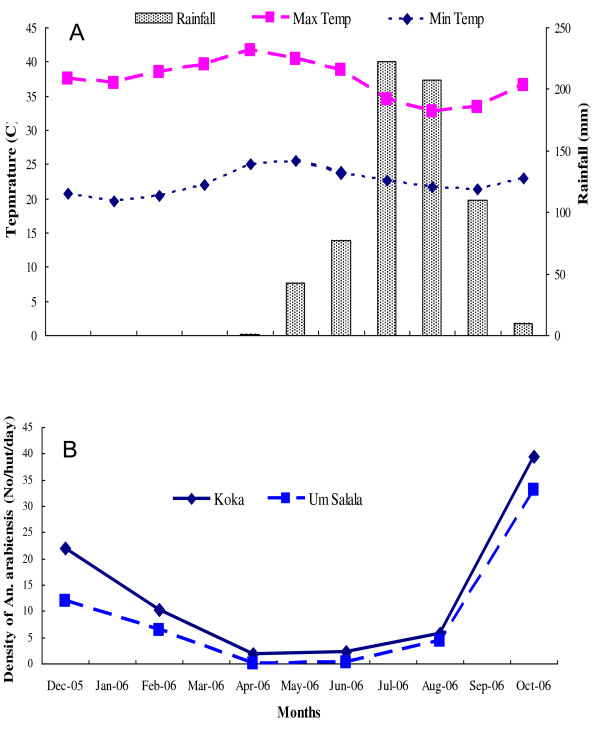
**Fluctuation of rainfall and temperatures (A) and densities of *An. arabiensis *(B) in the study villages, eastern Sudan, December 2005 - October 2006**.

This study was conducted in Koka and Um Salala villages (about 50 kilometers from each other), which are located on the eastern bank of the Rahad River, about 400 kilometers south-east of Khartoum (Figure 1). Um Salala is inhabited by the Massalit tribe who migrated from El-Geneina in Darfur State, Western Sudan, and settled along the Rahad River. The village of Koka is closer to the river, established 50 years ago and inhabited by the Hausa, an Afro-Asiatic speaking ethnic group originally from northern Nigeria. The inhabitants of the two villages live in African huts which are constructed of wood, bamboo and grass. The Rahad River is the main environmental feature in the area and is a tributary of the Blue Nile, originating in the Ethiopian highlands, west of Lake Tana. It flows more than 480 km northwest into the eastern part of Sudan to join the Blue Nile north to the town of Wad Medani. The river breaks into small ponds during the cool and hot, dry seasons (November - May) until the short rainy season in July - October during which a large flow occurs and floods the river. The ponds that are formed by the river become adequate breeding habitats for the principal malaria vector, *An. Arabiensis*. The land around the river is generally flat, but in many places it is interrupted by small seasonal streams and little ground surface water collection. The area is generally described by great seasonal fluctuation of climatic variables that can be distinguished in three main seasons; cool dry (November - February), hot dry season (March - June) and the rainy seasons (July - October). Climatic data collected from Gadaref meteorological station during the study period showed that the highest maximum temperature (41.7°C) was occurred in April and the lowest minimum temperature (19.7°C) in January. Rainfall peaked in July - August and the total amounts recorded during the study period were little (669.3 mm) (Figure 2A). *Plasmodium falciparum *is the predominant malaria parasite and its prevalence differed significantly by age group and being highest in under 5-year-olds [16]. In non- irrigated areas of eastern Sudan, a single peak of *An. Arabiensis *density was observed at the end of the short rainy season and then dropped gradually to disappear in the long, hot dry season [15].

### Mosquito sampling and identification

Mosquitoes were monitored in the two villages from a randomly selected sample of twenty houses once every two months. These 20 houses were fixed for adult samplings throughout the study period between December 2005 and October 2006. As the density of the vector *An. arabiensis *has been shown to be very low and with marked seasonality in the region [15], only the PSC method was used for sampling the adult vector [18]. In the field, collected mosquitoes were preserved in Petri dishes with moist filter paper and brought to the laboratory for further processing.

Mosquitoes were identified according to species on the basis of morphological characteristics [19]. A proportion of females of *An. gambiae *complex were identified to sibling species by polymerase chain reaction (PCR). DNA was extracted from mosquito legs and the PCR reaction conditions used were; denaturing (1 cycle for 5 minutes at 94°C), annealing (35 cycles; for 30 seconds at 94°C, for 40 seconds at 50°C, for 40 seconds at 72°C), and extension (1 cycle for 10 minutes at 72°C) [20]. Primers used were specific for *An. arabiensis*, *An. gambiae s.s*, *An. quadriannulatus *and *An. merus*, members of the *An. gambiae *complex. At the time of mosquito collection, the number of individuals who slept in each house the previous night was recorded.

### Sporozoite rate and human blood meal source determination

Female anopheline mosquitoes were recorded according to their abdominal status as unfed, freshly fed, half-gravid and gravid. The head and thorax of blood-fed and half gravid females of *An. gambiae *complex were separated from abdomen and stored separated in labeled vials containing 80% ethanol. The heads and thoraces of all anopheline mosquitoes were examined for the presence of *P. falciparum *circumsporozoite (CS) antigen [21]. Mosquitoes were ground in 50 μl of boiled casein containing IGPAL CA-630 and final volume brought to 250 μl with blocking buffer. Fifty microliters of the titrate was used in sporozoite enzyme-linked immunosorbent assays (ELISA). Positive reactions were assessed visually and each positive sample for sporozoite infection was retested for further confirmation [22]. The sporozoite rate was calculated by dividing the number of positive mosquitoes by total number of mosquito tested. The mosquitoes found to be positive for human blood meal were used to derive the human blood index. The ELISA procedure for blood meal source determination was adopted as described previously [23]. Briefly, each mosquito was ground in 50 μl of 0.01 M phosphate buffered saline (1x PBS), pH 7.4 and frozen at 20°C until tested. Mosquito triturate (50 μl) was diluted in PBS (1:50) and added to wells of polyvinyl chloride, U-shaped, 96-well microtitre plates. Four negative controls (unfed mosquitoes from the insectary) and one positive control were used. The plate was covered and incubated at room temperature for 3 hours. Each well was then washed twice with PBS containing 0.5% Tween 20, followed by the addition of 50 μl host specific conjugate (antihost IgG, H&L) diluted 1:2,000 or 1:250 for bovine in boiled casein containing 0.025% Tween20. The plates were incubated for one hour in room temperature, washed three times with PBS-Tw20 and 100 μl of ABTS (2, 2'-azino-di-[3-ethyl benzthiazoline sulfonate]), peroxidase was added to each well and the absorbance at 405 nm was determined with an ELISA plate reader 30 minutes after the addition of substrate. Reactions of dark green color were used as indicator for human blood presence. Samples were considered positive when the absorbance value exceeds the mean plus three times the standard deviation of the four negative controls.

### Entomologic inoculation rate (EIR)

EIR is a standard measure of transmission intensity and is expressed as the number of infective bites per person per unit time (e.g., per day, month , year). It was calculated by multiplying the human-biting rate by the proportion of sporozoite positive mosquitoes. The human biting rates (the number of biting mosquitoes per human- night), was calculated by dividing the total number of blood-fed and half-gravid mosquitoes caught in PSC catches by the number of persons slept in the house the night preceding collection and multiplied by the human blood index. The annual (ib/p/y) inoculation rate was derived by multiplying the daily EIR (ib/p/n) by 365.

### Data analyses

The human biting rates (bites/person-night), sporozoite rates (% of mosquitoes found ELISA-positive for CSP) and entomological inoculation rates (EIR; infective bites per person/night//person/year) were calculated for the different populations and seasons. Chi-square test was used to compare the proportions of mosquitoes with *P. falciparum *sporozoite among the different seasons and populations. Homoscedastic T - tests was used to examine the differences in means of *An. arabiensis *density, human biting rate, and the EIR between the two populations. Tukey-Kramer HSD test was applied to compare the means of these entomological indices among the different seasons. The association between *An. arabiensis *density and monthly total rainfall was examined using regression analysis. The analysis was conducted using JMP statistical software (JMP SAS Institute Inc. 2003).

### Ethical approval

Ethical approval for this study was obtained from the Institute of Endemic Diseases Ethical Committee and from the National Ethical Committee of Sudan.

## Results

### *Anopheles *species

A total of 1,402 adult female anopheline mosquitoes were sampled during the study period, of which 98% (n = 1374) were *An. gambiae *complex and 2% (n = 28) were *An. rufipes*. None of *An. rufipes *out of 13 females tested was found to have fed on human blood. A proportion of *An. gambiae *complex (n = 227) specimens from different seasons were identified by the PCR and all found to be *An. arabiensis*.

### Densities and human biting rates

Among *An. arabiensis *collected, the specimens from Koka and Um Salala accounted for 59.2% (n = 814) and 40.8% (n = 560) over the study period. This yields overall means of 13.57 and 9.33 female *An. arabiensis *mosquitoes/hut/day in Koka and Um Salala, respectively. The difference in the mean density between the two villages was not significant (*t *= 0.54, d.f. = 1, *P *= 0.60). Also, there was no statistically significant difference found between the means of human biting rates per person per night between Koka (mean = 2.34 b/p/n) and Um Salala (mean = 0.29 b/p/n) villages (*t *= 1.27, d.f. = 1, *P *= 0.23) (Table 1).

**Table 1:** Means of *An. arabiensis *density, human biting rate, sporozoite rate and entomological inoculation rate (EIR) in Koka and Um Salala villages, eastern Sudan, December 2005 to October 2006.

Village	**Density (female/hut)**^**1**^	HBR (bite/person)	CSP (%)	EIR (ib/p/n)
Koka	13.57 (1.20 - 25.93)^2^	2.34 (-0.2 - 4.88)	7.25 (3.39 - 11.10)	0.30 (-0.06 - 0.66)
Um Salala	9.33 (-3.03 - 21.70)	0.29 (-2.25 - 2.82)	0.79 (-3.07 - 4.65)	0.01 (-0.36 - 0.37)
*P Value*	0.601	0.232	0.025	0.231

^1^HBR: Human biting rate (bite/person/night), CSP: Sporozoite rate (No. CSP positive/total tested), EIR: Entomological inoculation rate (infective bite/person/night).

^2 ^Number between the two brackets are 95% confidence interval.

However, among the two sites, the density of *An. arabiensis *fluctuated evenly over the months, displaying with bimodal annual peaks (Figure 2B). The main peak density occurred in October, during which 48.8% and 59.1% of the total *An. arabiensis *collected in Koka and Um Salala, respectively. The corresponding human biting rates observed during this month were 10.12 and 1.07 b/p/n in Koka and Um Salala, respectively. A minor peak occurred in December and the average human biting rates were 2.58 and 0.4 b/p/n in Koka and Um Salala, respectively. The lowest numbers of *An. arabiensis *were sampled during the period of hot dry season (April - June) when the temperature was at its maximum, rainfall were little and the ponds around the river basin were almost dry (Table 2).

**Table 2:** Summary of surveys showed total number collected, biting rate (BR), human blood index (HBI), human biting rate (HBR), sporozoite rate (CSP) and entomological inoculation rate (EIR) of *An. arabiensis *in Koka and Um Salala, eastern Sudan, December 2005 - October 2006.

Month	Koka						Um Salala					
	**No. collected**	**BR**	**HBI (%)**	**HBR**	**% CSP (No)**	**EIR**	**No. collected**	**BR**	**HBI (%)**	**HBR**	**% CSP (No)**	**EIR**

Dec 05	219	3.20	80.46 (70/80)	2.58	14 (8/175)	0.360	119	0.500	80 (36/45)	0.40	1.1 (1/90)	0.004
Feb 06	102	1.30	67.35 (33/49)	0.88	0 (0/99)	0.000	64	0.300	61.54 (16/26)	0.19	1.59 (1/6)	0.003
Apr 06	19	0.04	60 (3/5)	0.02	8.3 (1/12)	0.002	0	0.000	0.00	0.00	0 (0)	0.000
Jun 06	22	0.37	83.33 (5/6)	0.31	5 (1/20)	0.015	3	0.000	0.00	0.00	0 (0/3)	0.000
Aug 06	58	0.20	65.22 (15/23)	0.13	2.17 (1/46)	0.003	43	0.100	62.5 (10/16)	0.06	0 (0/33)	0.000
Oct 06	394	11.03	91.7 (110/120)	10.12	14 (37/264)	1.416	331	1.300	82.19 (60/73)	1.07	2.05 (3/146)	0.022

BR: Biting rate (No. fed mosquito/No. sleeper), HBI: Human blood index (No. human blood positive/total tested), HBR: Human biting rate (bite/person/night), CSP: Sporozoite rate (No. CCSP positive/total tested), EIR: Entomological inoculation rate (infective bite/person/night).

This pattern of *An. arabiensis *fluctuation closely followed the rainfall and the subsequent recess of the Rahad River upon its fragmentation into disparate ponds of water that are distributed on the river bed during the hot and cool and dry seasons. This relationship between monthly total rainfall and the relative densities of *An. arabiensis *established after a two months time lag was shown to be statistically significant (Koka *r *= 0.79, d.f. = 1, *P *= 0.05; Um Salala, *r *= 0.88, d.f. = 1, *P *= 0.02).

During the different seasons, the highest density of the vector *An. arabiensis *(22.6 females/hut/day) was observed during the rainy season in Koka and the lowest one of 0.15 females/hut/day was recorded during the hot dry season in Um Salala. In both villages, high density occurred during the cool dry season generated by similar variations over the seasons (in Koka, *F*_*2,3 *_= 1.04, *P *= 0.45; in Um Salala, *F*_*2,3 *_= 1.20, *P *= 0.41). A similar pattern was also reported for the human biting rate and the variations observed over the seasons were not statistically significant in both villages (in Koka, *F*_*2,3 *_= 0.75, *P *= 0.54; in Um Salala, *F*_*2,3 *_= 0.91, *P *= 0.49) (Table 3).

**Table 3:** Means of *An. arabiensis *density, man biting rate, Sporozoite rate, human biting index and entomological inoculation rate (EIR) during the different seasons in Koka and Um Salala villages, eastern Sudan, December 2005 to October 2006.

Season	Koka				Um Salala			
	
	Density	HBR	CSP (%)	EIR	Density	HBR	CSP (%)	EIR
Cool dry (November - February)	16.05	1.73	2.92	0.18	9.15	0.29	1.31	0.004
Hot dry (March - June)	2.05	0.17	6.25	0.01	0.15	0.00	0.00	0.000
Rainy (July - October)	22.6	5.12	12.26	0.71	18.70	0.57	1.68	0.011
*P Value*	0.453	0.544	0.981	0.543	0.414	0.492	0.090	0.536

Density: female/hut, HBR: Human biting rate (bite/person/night), CSP: Sporozoite rate (No. CCSP positive/total tested), EIR: Entomological inoculation rate (infective bite/person/night).

### Sporozoite and entomological inoculation rates (EIR)

In Koka, equal *P. falciparum *sporozoite rates (CSP = 14%) were observed in the population of *An. arabiensis *during both peaks in October (37/264) and in December (8/175). In Um Salala, the minor peak of *P. falciparum *sporozoite rate (1.59%) occurred in February (Table 2). In both villages, the main peak of the EIR occurred in October (1.42 ib/p/n in Koka and 0.02 ib/p/n in Um Salala) and the minor one, in December (0.36 ib/p/n in Koka and 0.004 ib/p/n in Um Salala) (Table 2). The lowest mean EIR was reported during the hot and dry seasons in both Koka (0.01 ib/p/n) and Um Salala (0.0 ib/p/n). Overall, the EIR remained similar throughout the seasons for both populations (Koka, *F*_*2,3 *_= 0.75, *P *= 0.54; and Um Salala, *F*_*2,3 *_= 0.77, *P *= 0.54). This is in spite of the fact that there was a significant difference in the proportion of infected mosquitoes in Koka (*χ2 *= 14.00, d.f. = 2, *P *= 0.001) whereas this was not the case in Um Salala (*χ2 *= 4.815, d.f. = 2, *P *= 0.090). Likewise, the EIR was similar between the two villages (*t *= 1.28, d.f. = 1, *P *= 0.23) in spite of the difference observed in the CSP between the two village vector populations (*χ2 *= 11.21, d.f. = 1, *P *= 0.0014) (Table 2 &3).

While the observed average of the EIR was 0.30 ib/p/n,in Koka, it was 0.01 ib/p/n in Um Salala. (Table 1). The average EIR was also ranged from 0.01 ib/p/n during the hot dry season to 0.31 ib/p/n during the rainy season (Table 3). The combined average of the EIR calculated over the study period for the populations was 0.152 ib/p/n (95%CI, -1.0 - 9.1) and on average it would take approximately 192.7 days for an individual to receive an infective bite from *An. arabiensis*. The annual EIR estimated in the study area was 55.48 ib/p/y.

## Discussion

The ribosomal DNA-polymerase chain reaction has revealed that *An. arabiensis *was the only member of *An. gambiae *complex found in the study area. These results were similar to the previous observations from eastern Sudan [24,25]. This vector has shown to adopt better to hot and arid conditions than *An. gambiae s.s. *[26,27]; it appears to be distributed across Africa, predominantly in arid savannahs, in ecosystems similar to those found in the study area [28,29]. The other anopheline species reported in the study area was *An. rufipes *which showed non-human blood feeding behaviour similar to the bovine blood feeding observed in rice cultivation areas of central Kenya [30].

The present study showed that the number of infective bites due to *An. arabiensis *was fluctuated with bimodal annual peaks. The major biting peak occurred during the rainy season when 79% of the total infective bites took place. The minor biting peak occurred during the cool dry season in December. Similar patterns have been shown in the same region in a permanent irrigated area of eastern Sudan [31]. In general, the pattern observed in this area may be accounted for by seasonal rainfall, seasonal increase in the volume of the Rahad River and by temperature. In Sudan, recent studies showed that the fluctuation of seasonal density of *An. arabiensis *was generally fallowed rainfall [15]. However, in areas where irrigated agricultural or rivers are found, a minor seasonal peak density of *An. arabiensis *was confirmed during the cool dry season [31-35]. It is well established that in eastern Sudan, rainfall is the significant climatic variable in the transmission of the disease, whereas heavy rainfall has been confirmed to initiate epidemics [17]. In irrigated areas and along the river basins, a number of intermittent pools are persistent and maintain a relative low level of breeding activity of the vector *An. arabiensis *during the hot dry season [32,36]. In the present study, the role of the Rahad River could be further confirmed by the relative high, but not significant, densities observed in Koka, the village closer to the river relative to Um Salala. In sub- Saharan Africa, it has been documented that houses or villages near water bodies i.e. swamps, rivers or streams, had higher *Anopheles *density than those just a few hundred meters away from the rivers or streams [37-39].

The present study showed that the sporozoite rate determined in Koka was nine folds higher than Um Salala and that people living in Koka received 30 times higher infective biting rates than those resides at Um Salala (Table 1). This could be supported by the fat that marked variation in the prevalence of *P. falciparum *malaria has been observed between the two populations and the infection was high in Koka population [40]. Probably, this could be attributed to the high proportion of asymptomatic infections that has been shown previously among the Koka population during the hot dry season [40]. In fact, in the present study, more than 34% of the different in the sporozoite rate between the two villages was related to this period. In eastern Sudan, the asymptomatic infections of the dry season have been shown to demonstrate high proportion of gametocytes (40%), and this has been confirmed by the sensitive gametocyte-specific RT-PCR [41]. Based on mosquito feeding studies, it has been estimated that 28% of all transmission under setting of western Kenya is derived from asymptomatic gametocytes reservoir. This corresponded to 10-50 infectious bites per year, plenty to sustain a hyper or holoendemic pattern of malaria even if every symptomatic patient received highly effective treatment [42]. In general, in sub-Saharan Africa, it has been accepted that villages only a few kilometers apart can have EIRs differing by several ten times [2,43-46].

Overall, the combined mean annual *P. falciparum *EIR in the study area was 55.48 ib/p/y. This appeared to be greater than 2-3 ib/p/y that have been shown in non irrigated areas of eastern Sudan [15]. However, the mean was still bellow the level of high endemic areas, where annual EIRs could be more than 700 ib/p/y in a period for more than six months [47]. The pattern of transmission observed in the study area is likely perennial and moderate rather than seasonal and low and had typical fluctuation to that observed in areas of permanent irrigation system [17]. These data suggests that, rainfall and riverine breeding sites are the main factors driving transmission pattern in the area whereas asymptomatic infection is likely to increase transmission intensity. Investigating spatial distribution of the most productive habitats is vital for targeting larval control. Research should continue to establish a safety and cost effective protocol for quantifying and treating the asymptomatic infection [48]. This appears to be more important in order to reduce the overall transmission, particularly when plan of malaria control move towards elimination.

## Conclusion

Given the observed bimodal annual peaks and active transmission during the hot dry season and the fact that the vector *An. arabiensis *has shown exhibited exophilic feeding behaviour [31] and knock down resistance (kdr) mechanism [25], none adulticides vector interventions such as larval control or source reduction should be integrated with the current vector control strategies of mass distribution of ITNs which has been shown confer less protection against exophagic vectors [49]. In these dry savannah areas of semi-desert environments, such larval control program is more appropriate to be operated during the dry seasons when the distributions of vector breeding sites are limited to certain areas [50].

## Competing interests

The authors declare that they have no competing interests.

## Authors' contributions

YEH performed data management and statistical analyses, provide results interpretation, drafted and write up the manuscript, MME supervised data collection and carried out the laboratory analyses, IME & MI advised on the design and implementation of the study, EJK performed analysis, reviewed and finalized this paper for publication. All authors read and approved the final manuscript.
